# Growth Hormone Receptor Antagonism Extends Lifespan

**DOI:** 10.1111/acel.70697

**Published:** 2026-09-06

**Authors:** Edward O. List, Darlene E. Berryman, Grace S. Lach, Delaney F. Minto, Keeley Weese, John J. Kopchick

**Affiliations:** ^1^ Institute of Molecular Medicine and Aging, Heritage College of Osteopathic Medicine Ohio University Athens Ohio USA; ^2^ The Department of Biomedical Sciences, Heritage College of Osteopathic Medicine Ohio University Athens Ohio USA

**Keywords:** antagonist, GH, GHA, GHR, GHRA, growth hormone, growth hormone receptor

## Abstract

Interventions that disrupt growth hormone (GH) action are recognized as some of the most potent methods for extending lifespan. Accordingly, GH receptor antagonists (GHA) represent potential therapeutics to improve healthspan. Somavert (Pegvisomant for injection), used for treating patients with acromegaly, is currently the only FDA approved GHA. This drug was based on our laboratory's early 1990s discovery that mutating a codon for a conserved glycine—at position 119 in bovine GH or 120 in human GH—to a variety of amino acids, including lysine, ultimately converted GH from an agonist to antagonist. Since Pegvisomant has poor affinity to rodent GHR, it has not been tested for its ability to extend lifespan in rodents. To address this gap, we evaluated survival in GHA transgenic mice, a mouse line that played a crucial role in the discovery and development of Pegvisomant and has been maintained in our lab since 1991. While a prior study with several limitations failed to detect lifespan extension in GHA mice, our current study addressing these limitations shows that both median and maximal lifespan were significantly increased in male (*p* = 0.044; *p* = 0.0037) and female GHA mice (*p* = 2 × 10^−8^; *p* = 9 × 10^−6^), with maximal lifespan extended by 186 and 265 days, respectively. Analysis of an independent cohort of 2‐year‐old mice revealed that GHA males and females were less frail with enhanced grip strength despite increased adiposity. These findings demonstrate for the first time that GH antagonism can improve health and extend lifespan.

## Graphical abstract

This study provides proof of concept that GH antagonism in GH‐producing mice extends lifespan, supporting further investigation of GH antagonists as potential anti‐aging interventions in animals and humans.

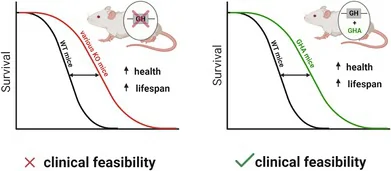

## 1 Introduction/Results/Discussion

A large and growing number of studies in mice have shown that genetic alterations that reduce or eliminate growth hormone (GH) action are among the most potent interventions for extending lifespan (Bartke and Quainoo 2018; Brown‐Borg 2015; List et al. 2024). Numerous inactivating mutations in members of the GH/IGF‐1 axis have been shown to extend lifespan; however, mutations that specifically disrupt GH or its production—thereby reducing both GH action and IGF‐1—result in some of the largest reported increases in lifespan in laboratory mice (i.e., GH, GHR, GHRH, GHRH‐R, Pou1f1 and Prop1 null mice). According to the Rodent Aging Interventions Database (https://www.levf.org/projects/raid), the longest median lifespan extension for a group of laboratory mice (+625 days; representing a 109% increase) resulted from a mutation in the Prop1 gene (Brown‐Borg et al. 1996). While isolating the specific contribution of GH is difficult in Prop1 deficient mice since they lack multiple pituitary hormones, administering high doses of porcine GH in early life reduces extreme longevity in these mice, implicating GH as the key responsible hormone (Sun et al. 2017). Furthermore, the world's longest lived laboratory mouse, which received the Methuselah mouse prize, resulted from a null mutation in the GHR gene and lived 1 week shy of 5 years (Pilcher 2003). This mouse line was generated in our laboratory and aged in the Bartke laboratory after backcrossing into a mixed genetic background. Our laboratory subsequently demonstrated that this effect is so potent that removal of GHR in later life (Duran‐Ortiz et al. 2021; Junnila et al. 2016) or even in a single tissue (List et al. 2015, 2022) is sufficient to elicit a measurable extension in lifespan. While these and other studies suggest that inactivating genes involved in GH action promote healthy aging, methods used to generate these mice are not feasible to replicate in the clinic. Accordingly, Pegvisomant (the only FDA approved GHR antagonist) has been proposed to have significant potential as an anti‐aging therapeutic (Longo et al. 2015; Milman et al. 2016). Unfortunately, since Pegvisomant has poor affinity for mouse GHR, it has not been tested for its ability to increase lifespan in rodents. To circumvent this limitation, the current study used transgenic mice that express GHA as a surrogate for Pegvisomant treatment, and survival and select health parameters were determined.

As for Pegvisomant, studies from our laboratory in the early 1990s led to the discovery that substitution of a highly conserved glycine residue in GH generates a potent GH receptor (GHR) antagonist (Chen, White, et al. 1991; Chen, Wight, Chen, et al. 1991; Chen et al. 1990). These findings ultimately led to the development of Pegvisomant (Kopchick et al. 2014). Importantly, the early clinical studies with Pegvisomant established GH antagonism as a practical and well‐tolerated method for reducing GH action. The favorable safety profile of GH receptor antagonism has generated substantial interest in the endocrine field and has led to numerous efforts to develop next‐generation GH antagonists and other strategies to safely reduce GH action in humans (Basu et al. 2025; Lu et al. 2019; Wang et al. 2023). As a result, GH antagonism represents a clinically relevant approach for determining whether the longevity benefits associated with reduced GH signaling can be achieved without genetic manipulation of the GH/IGF‐1 axis.

Our laboratory has continued to maintain the original bovine GHA line generated in 1991 with glycine 119 mutated to lysine (G119K), and its characterization has served as a valuable tool for evaluating the physiological effects of GHR antagonism. This line has been backcrossed more than 20 generations and maintained in a C57BL/6J background to match the genetic background of our other GH‐modified mouse lines for comparison (Berryman et al. 2004; Coschigano et al. 2003). GHA mice have a~70% reduction in serum IGF‐1 levels (Chen et al. 1995) and remain smaller in size than WT counterparts (Coschigano et al. 2003). These mice also have a marked decrease in lean mass and a significant increase in fat mass, largely contained in the subcutaneous depot but more broadly distributed than that found in GHR^−/−^ mice, with this phenotype being more pronounced in males (Berryman et al. 2014).

Important to the current study, GHA mice show signs of healthy aging as they exhibit improved cognitive function (Basu et al. 2017), reduced fibrosis (Householder et al. 2018), reduced cancer susceptibility (Basu et al. 2022; Pollak et al. 2001), and protection from osteoarthritis (Zhu et al. 2023) and kidney damage (Chen et al. 1995). Remarkably, despite marked obesity, they are protected from hyperinsulinemia and glucose intolerance when placed on a high fat diet (Yang et al. 2015). However, an initial scan of our colony back in 2003 failed to detect extended longevity in GHA mice (Coschigano et al. 2003). This finding was surprising since it contradicted the consensus view in the literature that reduced GH action in mice extends lifespan. However, this initial study had several limitations that are addressed here. More specifically, the previous study was not a formal aging study as mice were not specifically set aside for aging, but rather lifespan was determined by scan of deaths in a relatively small number of mice (*n* = 22–33) from the breeding colonies; thus, breeders and non‐breeders were included. Furthermore, survival data showed a distinct trend towards increased lifespan—especially in females—but were compared using ANOVA rather than a more commonly accepted test such as Log‐Rank.

**FIGURE 1:**
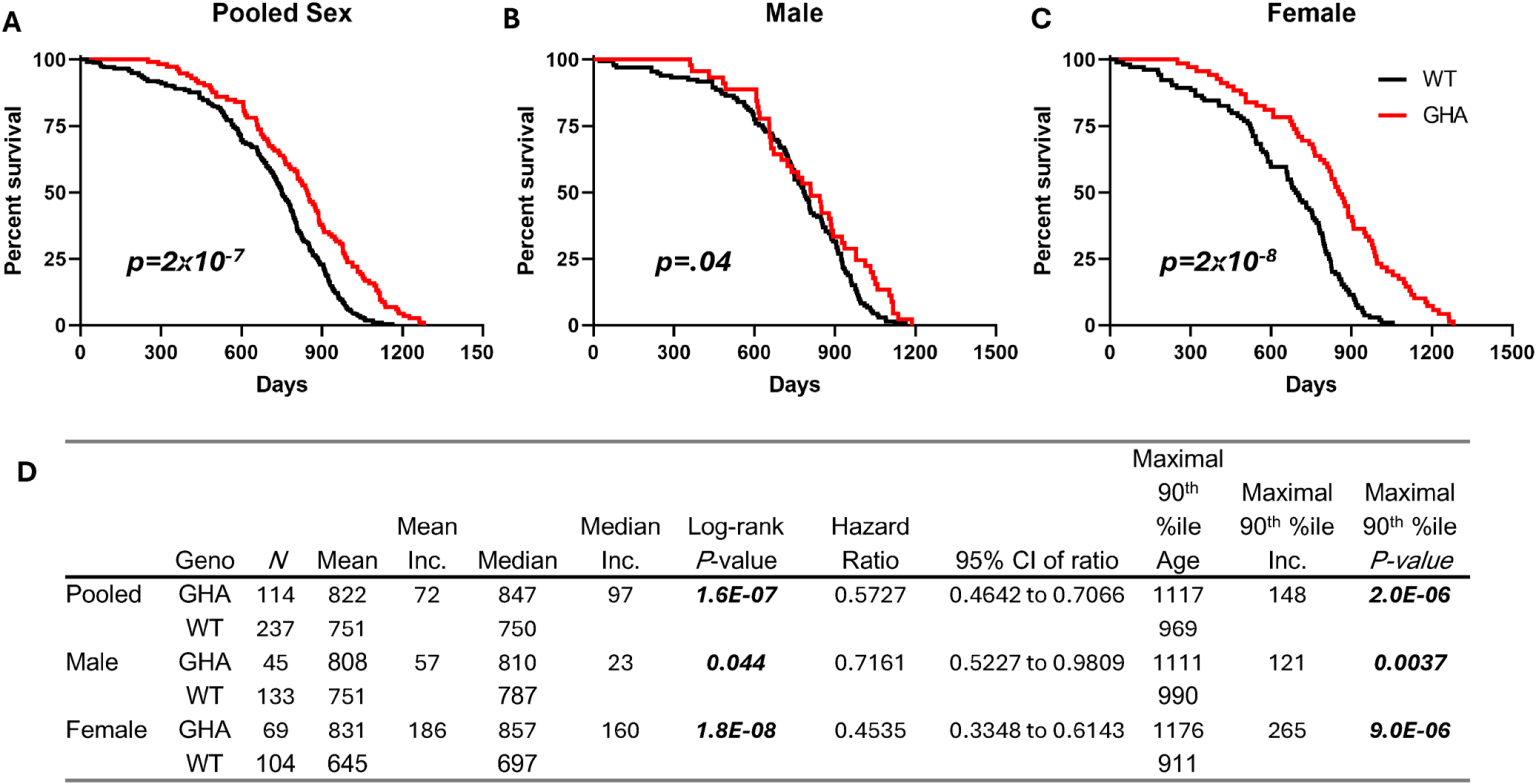
Transgenic mice expressing a GH antagonist are long‐lived. Kaplan–Meier curves comparing survival between WT (black) and GHA (red) mice are shown for (A) pooled sex, (B) males, and (C) females. (D) A corresponding survival statistics table including numbers of mice per group, mean and median survival, log‐rank *p*‐value, and maximal lifespan calculations are shown for each of the three comparisons. Mean, median, and maximal values as well as increases in these measures are expressed in days. GHA mice used in this study have been characterized and described (Berryman et al. 2004; Chen et al. 1995; Chen, Wight, Mehta, et al. 1991; Coschigano et al. 2003). Briefly, GHA mice in a C57Bl/6J background were generated by pronuclear microinjection of a bovine GH minigene driven by mouse metallothionine‐1 promoter/enhancer; thus, the transgene expression drives expression in a variety of tissues with liver having the greatest expression. The transgene contains a mutation in the third alpha helix resulting in a glycine at position 119 being replaced by a lysine. The plasmid used was pBGH10∆6, which contains the complete coding region of bGH and intron A. Serum bovine GHA level was previously determined to be 5.0 μg/ml resulting in a growth ratio at 2 months of 0.59 of wild type body weight (Chen, Wight, Mehta, et al. 1991). Animal husbandry and estimation of death were performed as previously described (List et al. 2022). Of the 351 mice in the study, 298 were found dead, 37 were euthanized with advanced ulcerative dermatitis, 16 mice were euthanized due to poor health other than ulcerative dermatitis. Survival curves were compared using the log‐rank test. All significance tests utilized for survival are based upon the two‐tailed at *p* < 0.05. For evaluation of maximal survival, a Fisher exact test at 90% survival was conducted.

**FIGURE 2:**
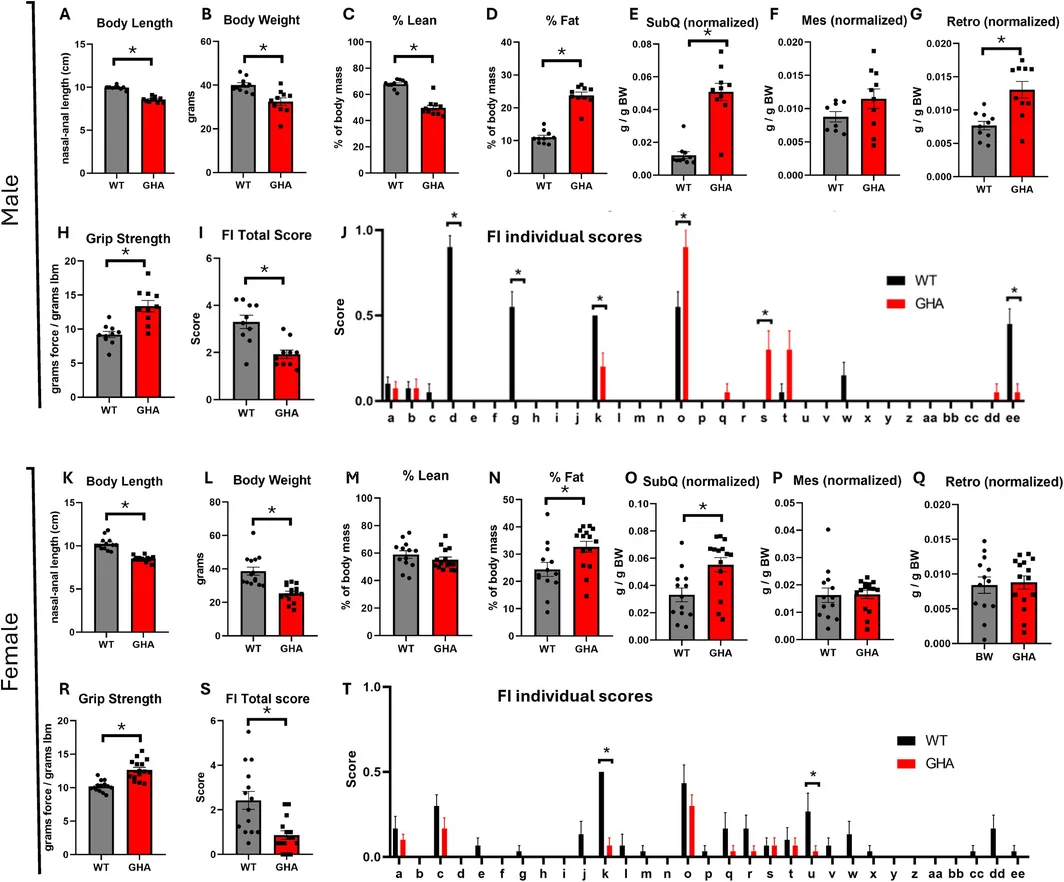
Cross‐sectional analysis. A separate cohort of 2‐year‐old male (top half) and female (bottom half) GHA (red) and WT (gray/black) mice not part of the aging study were used for cross‐sectional analysis as described (List et al. 2022). (A, K) body length, (B, L) body weight, (C, M) % lean mass, (D, N) % fat mass, (E, O) subcutaneous WAT, (F, P) mesenteric WAT, and (G, Q) retroperitoneal WAT. WAT depot weights were normalized to body weight. (H, R) grip strength and (I, S) clinical frailty index total score, and (J, T) individual test scores as defined (Whitehead et al. 2014), which includes (a) temperature, (b) weight, (c) alopecia, (d) fur color, (e) dermatitis, (f) whiskers, (g) coat, (h) tumors, (i) abdomen, (j) kyphosis, (k) tail, (l) gait, (m) tremor, (n) grip, (o) body condition, (p) vestibular disturbance, (q) hearing, (r) cataracts, (s) eye discharge, (t) microphthalmia, (u) corneal, (v) vision, (w) menace, (x) nasal, (y) malocclusions, (z) rectal, (aa) vaginal/penile, (bb) diarrhea, (cc) breathing, (dd) grimace, and (ee) piloerection, are presented. Means ± SE are shown, and significance was determined by a Student *t*‐test. A *p* value of < 0.05 was considered statistically significant.

Because of the limitations in our 2003 study and its contradiction to the consensus, we saw a critical need for a formal aging study with sufficient power to better determine if GH antagonism can indeed extend longevity. In the current study, we evaluated survival using 351 GHA and WT mice (*n* = 45–133) specifically set aside for survival analysis (i.e., no breeders were included, and mice were used for no other measures other than survival). In all groups (i.e., pooled sex, male only, and female only), lifespan was significantly increased for GHA mice (see Figure 1). Specifically, mean lifespan was increased 28.8% (186 days) in GHA females and 7.6% (57 days) in GHA males; median lifespan was increased 23.9% (160 days) and 2.9% (23 days), and maximal lifespan was increased by 29.1% (265 days) and 12.2% (121 days) in GHA females and GHA males, respectively. It should also be noted that while both sexes of GHA mice lived significantly longer, the improvement seen in females was more robust than males (186 vs. 57 days increase in mean lifespan, respectively). Accordingly, evaluating the potential contribution of sex hormones in future studies is warranted. Based on these exciting findings, we examined a separate cohort of 2‐year‐old mice to evaluate frailty, grip strength, body composition and fat distribution. Notably, both male and female GHA mice had superior grip strength and reduced frailty scores. This improvement occurred despite increased body fat, which was primarily sequestered in the subcutaneous (both sexes) and retroperitoneal (male only) depots (see Figure 2). It is important to note that the mice in this study were in a C57BL/6J inbred background—a strain that typically shows a diminished effect on longevity interventions due to lack of hybrid vigor. Because of this, many in the aging field, including the National Institute of Aging sponsored Interventions Testing Program, conduct aging studies in mixed genetic backgrounds. Thus, these improvements in lifespan and frailty are impressive given that they were obtained in a C57BL/6J inbred background. We are currently performing additional aging studies on GHA mice in a mixed background to further evaluate the anti‐aging potential of GHA.

The mechanisms underlying lifespan extension and reduced frailty in GHA mice are presumed to overlap with other well‐characterized mouse lines with reduced GH action (e.g., GHR^−/−^ mice, Snell, Ames, Lit/Lit etc.) though to a lesser extent, as GHA mice retain partial GH signaling through antagonism rather than null mutation. This distinction is important in that GH is a pleotropic hormone—playing an important role in numerous physiological functions; thus, complete removal of GH action from inception via genetic mutation is not desirable in humans. Collectively, these other mouse lines with reduced GH action show improved metabolic health—enhanced insulin sensitivity, elevated adiponectin, reduced tissue inflammation, and a greater metabolic flexibility (Bartke and Quainoo 2018; Brown‐Borg 2015; List et al. 2024) despite obesity, a seemingly paradoxical but highly reproducible “healthy obese” phenotype observed across multiple GH‐deficient and GH‐resistant models [as reviewed (Berryman and List 2017; Kopchick et al. 2020)]. These mice also exhibit exceptional cancer resistance (Ikeno et al. 2009). At the cellular and functional levels, they demonstrate reduced adipose senescence, attenuated immunosenescence, decreased insulin exposure and mTOR signaling, improved glucose homeostasis, AMPK activation, and enhanced antioxidant defenses, alongside preserved physical function, cognition, and stress resilience into late life (Bartke et al. 1998; Bartke and Quainoo 2018; Bashir et al. 2026; Brown‐Borg 2015; Junnila et al. 2013; List et al. 2024; Stout et al. 2014). Furthermore, while circulating GH levels decline with age, recent studies have identified that local GH production can increase in aging tissues and tumors (Chesnokova and Melmed 2022), meaning that attenuation of GH action in later life remains a viable gerotherapeutic. Importantly, GH is a component of the senescence‐associated secretory phenotype or SASP, suggesting that autocrine and paracrine GH signaling may remain elevated despite reduced endocrine GH (Chesnokova et al. 2013). These observations provide a rationale for targeting GH action later in life.

While substantial evidence indicates that reduced GH action can promote healthy aging and extend lifespan, the relationship between the somatotropic axis and health is complex. Severe loss of GH action in humans and rodents can be associated with physiological impairments involving multiple organs, including heart, kidney, pancreas, skeletal muscle and adipose tissue; restoration of GH axis signaling can improve some of these deficits (Ho et al. 2023; Milioto et al. 2026). For example, individuals with severe GH deficiency can exhibit reduced cardiac mass and exercise capacity, both of which can improve with GH replacement therapy (Milioto et al. 2026). Likewise, humans with GH deficiency have reduced glomerular filtration rate and renal plasma flow with GH‐replacement therapy restoring both in some but not all studies (Caidahl et al. 1994; Hoffman et al. 1996; Jorgensen et al. 1989). Further complicating this discussion, it has been shown that some “structural” changes seen with low GH signaling (e.g., reduced islet size or increased adiposity) do not necessarily translate into overt dysfunction under basal conditions. For example, congenital lack of GH action in mouse models results in reduced pancreatic islet size and β‐cell mass (List et al. 2019; Liu et al. 2004). However, these rodent models are characterized as glucose intolerant but insulin sensitive. Thus, these animals have excellent glycemic control under normal conditions, but when challenged with a large bolus of glucose (during a GTT), they have difficulty clearing excess glucose (List et al. 2019; Liu et al. 2004). Likewise, increased adiposity in these models is frequently accompanied by preserved metabolic health. Thus, structural changes alone should not be assumed to reflect physiological dysfunction. Overall, both beneficial and adverse outcomes have been reported with severe GHD, which need to be considered when translating these results in mice to humans.

In conclusion, numerous studies in mice demonstrate that genetic mutations that eliminate GH action dramatically increase lifespan and healthspan. This has sparked significant interest in using agents that attenuate GH/IGF‐1 signaling to target aging and age‐related diseases in humans (Longo et al. 2015; Milman et al. 2016). However, mutating genes involved in GH regulation is not feasible in humans as a therapeutic option. Furthermore, clinical conditions associated with severe GH deficiency do not always show health benefits. Thus, the importance of this study lies not in reaffirming that removal of GH action in animals is advantageous for healthspan and lifespan but rather in providing the first proof‐of‐concept that reducing GH action via GH antagonism in GH producing mice can extend lifespan. While both approaches converge on reduced GH action, the underlying methodology for this reduction is distinct and clinically achievable. Importantly, this study establishes a foundation for future studies in animals and, ultimately, humans to determine whether GH antagonists, which can be provided therapeutically at different doses and different times of life, represent a viable anti‐aging intervention.

## Author Contributions

Edward O. List, Darlene E. Berryman, and John J. Kopchick conceptualized the study. Edward O. List and Darlene E. Berryman wrote the manuscript. Edward O. List analyzed the data, performed statistics, and created graphs. Grace S. Lach maintained the colony and measured body composition. Delaney F. Minto measured grip strength and frailty. Keeley Weese assisted with the organization of death records. All authors commented and approved of the manuscript.

## Funding

This work was supported by National Institutes of Health grant R01AG059779, the State of Ohio's Eminent Scholar Program that includes a gift from Milton and Lawrence Goll, and the AMVETS.

## Conflicts of Interest

The authors declare no conflicts of interest.

## Supporting information


**Data S1:** acel70697‐sup‐0001‐Supinfo.docx.

## Data Availability

The data that support the findings of this study are available on request from the corresponding author. The data are not publicly available due to privacy or ethical restrictions.
