# Supplementary material for: Growth Hormone Receptor Antagonism Extends Lifespan
Source: Aging Cell. 2026 Sep 6;25(9):e70697. doi: 10.1111/acel.70697 (PMC13546677; doi:10.1111/acel.70697)
Supplement: Supplementary file 1 [file ACEL-25-e70697-s001.docx]

**Supporting Information - Methods**

**Animals/Housing.** GHA mice used in this study have been characterized and described (Berryman et al. 2004; N. Y. Chen et al. 1995; W. Y. Chen, Wight, Mehta, et al. 1991; Coschigano et al. 2003; Pollak et al. 2001). All mice were bred at Ohio University (OU) and weaned into permanent housing no earlier than age 21 days and no later than age 28 days. At weaning, ear notching was performed for mouse identification, and a portion (< 1 mm) of the distal tail was removed for genotyping. Mice were housed 3 or 4 per cage in ventilated microisolator cages using one-quarter-inch corn cob bedding (Bed O Cobs, produced by The Andersons) and given *ad libitum* access to water and rodent chow (ProLabRMH 3000; 14% of energy from fat, 60% from carbohydrates, and 26% from protein; PMI Nutrition International). The cages were maintained in a temperature- (22 ± 2 °C) and humidity-controlled room and exposed to a 14-hour light, 10-hour dark cycle. Cages and bedding were changed once per week. The microbial status of the room was specific-pathogen free as determined by quarterly serology and semiannual parasitology reports from sentinel animals placed in used bedding. All procedures were approved by the OU Institutional Animal Care and Use Committee and fully complied with all federal, state, and local policies.

**Estimation of Age of Death.** Mice were examined daily for signs of poor health and euthanized if considered by an experienced technician unlikely to survive more than a week. A mouse was considered moribund if exhibiting more than one of the following signs: large or bleeding tumor, prolapsed anus or vagina, inability to eat or drink, severe lethargy, rapid weight loss over 1 or more weeks, severe balance or gait disturbance, or advanced dermatitis (ulceration larger than 1 cm^2^ that does not resolve with trimming of nails). Of the 351 mice in the study, 298 were found dead in the cage (176 WT and 77 GHA), 37 were euthanized with advanced ulcerative dermatitis (28 WT and 9 GHA), and 16 mice were euthanized due to poor health described above other than ulcerative dermatitis (12 WT and 3 GHA).

**Grip strength, frailty,** **body composition, body length, and white adipose tissue depot mass in 2-year-old mice.** A separate cohort of 2-year-old male GHA and control mice not part of the aging study was used for 2-year measures. Grip strength and frailty determination were performed as described (List et al. 2022; Whitehead et al. 2014). Body composition was determined using a Bruker Minispec mq NMR analyzer (The Woodlands, TX, USA). One week after measuring frailty and grip strength, the mice were euthanized, nasal-anal body length was measured, and tissues collected and weighed.

**Statistical analysis.** Unless otherwise stated, means ± SE are presented in the figures, and significance was determined by a Student t-test. A P value of < 0.05 was considered statistically significant. Survival curves were compared using the log-rank test. All significance tests utilized for survival are based upon the two-tailed log-rank test at P < 0.05. For evaluation of maximal survival, a Fisher exact test at 90% survival was conducted.
